# Analysis of the nystagmus characteristics of cupula diseases: A case report

**DOI:** 10.1097/MD.0000000000028211

**Published:** 2022-01-07

**Authors:** Zhaoxia Wang, Yang Zhang, Qiang Guo, Ying Lin, Juan-Juan Li

**Affiliations:** a Department of Otolaryngology, Longgang E.N.T. Hospital & Shenzhen Key Laboratory of E.N.T., Institute of E.N.T. Shenzhen, China; b Aerospace Balance Medical Center, Chinese PLA Air Force Medical Center, Beijing, China; c Department of Otolaryngology & Head and Neck Surgery, Xijing Hospital, Air Force Medical University, Xi’an, Shaanxi, PR China.

**Keywords:** case report, cupula, direction-changing positional nystagmus, vertigo, zero plane

## Abstract

**Introduction:**

Clinically, there is a kind of patients with positional vertigo or dizziness, which occurs when they turn left or right, look down or up, lie down or sit up. With a long duration and varying frequency, it is not consistent with the manifestations of benign paroxysmal positional vertigo (BPPV). In addition, the persistent geotropic direction-changing positional nystagmus (PG-DCPN) was observed in a supine head-roll test.

**Patient concerns:**

With no apparent trigger for visual rotation and a sense of self instability, an 81-year-old female patient had suffered from vertigo for 3 days. The vertigo occurred every day, lasting several minutes each time, and associated with head movements and changes in body position. In a supine head-roll test, it appeared persistent geotropic direction-changing positional nystagmus for a long time, without latency, fatigability and in the presence of 3 zero planes.

**Diagnosis:**

Light cupula.

**Interventions:**

Difenidol hydrochloride 25 mg orally 3 times/day for 2 weeks and betahistine hydrochloride 12 mg orally 3 times/day for 1 month were administered.

**Outcomes:**

After 1 month of treatment, the patient's vertigo symptoms disappeared. And in the supine head-roll test, the persistent geotropic direction-changing positional nystagmus disappeared.

**Conclusion:**

We report the characteristics of nystagmus produced in a typical patient with light cupula during the supine head-roll test. After reviewing the relevant literatures, we believe that a simpler method can be used to identify canalolithiasis and cupula disease, to distinguish light and heavy cupula, and to determine the pathological semicircular canal to which the lesion belongs.

## 1 Introduction

We clinically describe a new class of patients with positional vertigo or dizziness. The clinical characteristics of these patients are very similar to those of benign paroxysmal positional vertigo (BPPV). However, the duration of positional vertigo is comparatively long and the nystagmus performance in the positional experiment does not conform to typical BPPV. In a head roll test, persistent geotropic direction-changing positional nystagmus (PG-DCPN) can be observed. The duration of nystagmus is comparatively long, with no incubation period and without any fatigue. In addition, the direction of the nystagmus can change when the zero plane is exceeded. The nystagmus in otoliths has a short incubation period as well as fatigue.

“Light cupula” is an emerging concept introduced by Shigeno et al to explain this variant of PG-DCPN.^[1,2]^ The incidence rate of light cupula was 4.9% in patients diagnosed with BPPV (a misdiagnosis in these patients) and 14.2% in patients diagnosed with DCPN.^[3]^ The rate and frequency of light cupula recurrence are higher than those of canalolithiasis and cupulolithiasis.^[4]^ The mechanism underlying the light cupula remains unclear, and the “lighter cupula,”^[5–8]^ “light debris,”^[9–11]^ and “heavier endolymph” hypotheses^[12–15]^ have not been confirmed. To date, no clear diagnostic criteria for light cupula have been presented. The clinical features reported in the literature include^[8,14,16]^:

1. weak spontaneous nystagmus on the healthy side in the sitting position;
2. PG-DCPN nystagmus without incubation period in the roll test;
3. disappearance of the nystagmus after the head is turned to the affected side about 15 to 30 ° in the supine position (second zero plane);
4. horizontal nystagmus points to the affected side in the bow test, and the healthy side in the lean test;
5. horizontal nystagmus toward the healthy side in the supine position;
6. a poor reset effect in HC-BPPV;
7. exclusion of migraine, BPPV, Meniere's disease, central vertigo, and other diseases.

Most researchers in the field agree that light cupula is not an independent disease. Instead, the light cupula is a pathological state of receptors of the external vestibular system receptors with multiple unknown etiologies. Thus, the light cupula can be described as a clinical syndrome (or syndrome). Here, we report a case of light cupula, review the literature, analyze the characteristics of cupula and DCPN, and summarize their key diagnosis points.

## 2 Case presentation

We present an 81-year-old woman who had visual rotation without obvious inducement. This instability (which began three days earlier) was related to changes in head movement and body position. She recounted that visual rotation was persistent in both standing and lying positions, which was also accompanied by continuous dizziness. No cochlear or neurological problems were reported. No obvious abnormality was found in the general examination, the nervous system examination, the tracking test, and the scanning test. No staring nystagmus or eye deviation was found. No acute disease was found on MRI or DWI. Although the patient had a history of hypertension and coronary heart disease, she had no history of episodes of vertigo, tinnitus, or hearing loss.

## 3 Results of positioning test

### 3.1 Roll test

1. Left-sided nystagmus with a slow angular velocity SPV of 7.8°/s could be observed when the patient was in the supine position (Fig. 1    A).
2. A turn of her head 90° to the right could induce a right nystagmus (geotropism nystagmus). It was very fast and abrupt (no incubation period), with attenuation, and was of long duration (>5 minutes), showing an initial SPV of the nystagmus of 5.2°/s. After reaching the maximum nystagmus 40 second later (SPV of 25.8°/s), the nystagmus gradually decreased. After 5 minutes of observation, the nystagmus had attenuated to 7.0°/s, and the mean value of the slower angular velocity SPV was 24.2°/s (Fig. 1    B).
3. In the process of turning her head slowly to the left from the 90° position to the right, a continuous right nystagmus could be observed. The intensity of the nystagmus gradually decreased, and the nystagmus disappeared at the 30° position to the right (Fig. 1    C; second zero plane). After a further turn of the head to the left to reach the midline, a faint left nystagmus could be seen, with a slow angular velocity SPV of 3.9°/s.
4. A turn of her head 90° to the left could induce a left nystagmus (geonystagmus). This nystagmus showed no fatigue, attenuation, and was long-lasting (>5 minutes), showing an initial SPV of the nystagmus of 24.6°/s. After reaching the maximum nystagmus 14 second later (SPV of 38.1°/s), the nystagmus gradually decreased. After 5 minutes of observation, the nystagmus had attenuated to 6.4°/s, and the mean value of the slower angular velocity SPV was 9.2°/s (Fig. 1    D).
5. In the process of turning her head slowly to the right from the 90° position to the left, a continuous left nystagmus could be observed. The intensity of the nystagmus gradually decreased, while the nystagmus and its direction remained.

**Figure 1:**
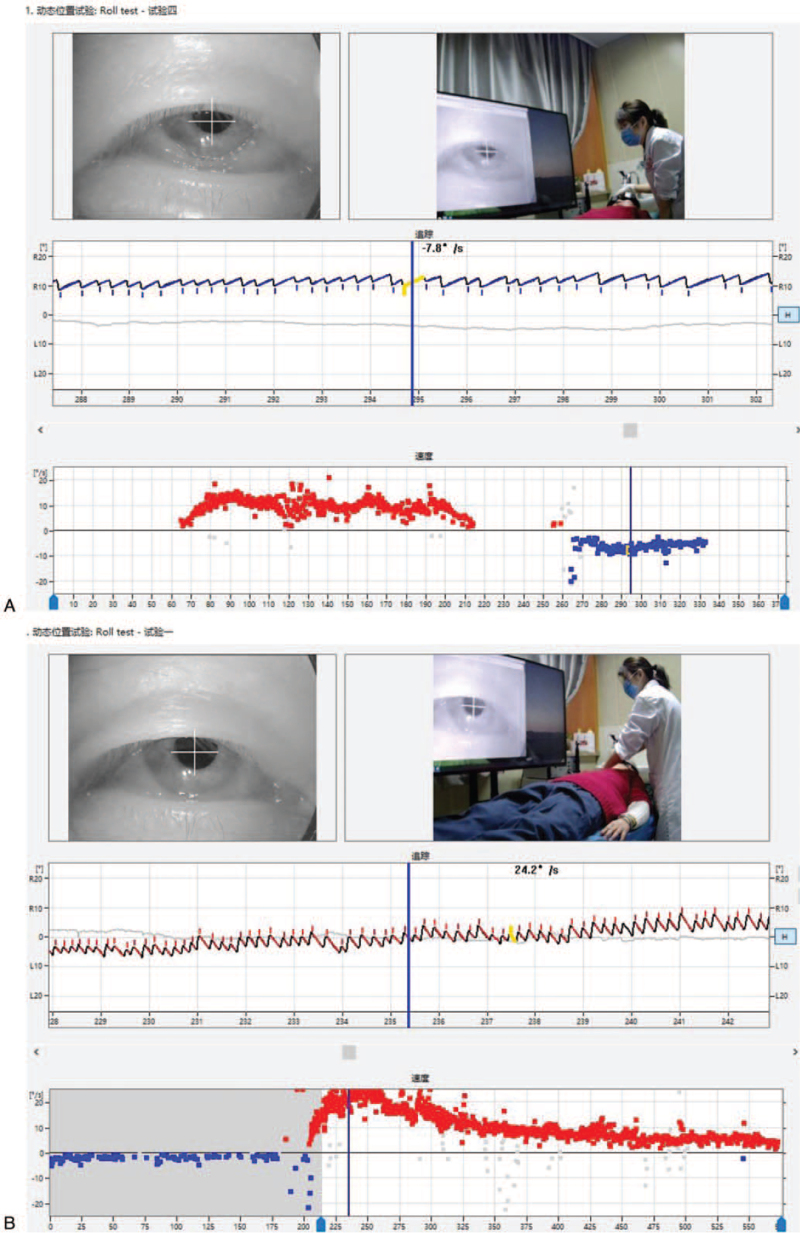
(A) A left horizontal nystagmus with an SPV of approximately 7.8°/s could be observed in the supine position. (B) In the Roll Test, a 90° rotation of the head to the right side induced sustained horizontal right nystagmus with an SPV of 24.2°/s. (C) With the head turned 15 to 20° to the right in the supine position, no nystagmus (second zero plane) was observed. (D) In the Roll Test, with the head turned 90° to the left, sustained horizontal left nystagmus was observed with an SPV of 9.2°/s. (E) A horizontal left nystagmus with an SPV of approximately 1.5°/s could be observed in the sitting position. (F) A horizontal right nystagmus with an SPV of 14.1°/s could be observed when the head was lowered 90° down in the sitting position. (G) The nystagmus disappeared (first zero plane) when the head was lowered 30° down in the sitting position. (H) A horizontal right nystagmus with an SPV of 22.6°/s could be observed in the prone position. (I) When the head was turned 15 to 30° to the right in the prone position, the direction of the nystagmus changed from right to left (reflecting the third zero plane). However, the actual plane of the nystagmus could not be found.

**Figure 1 (Continued):**
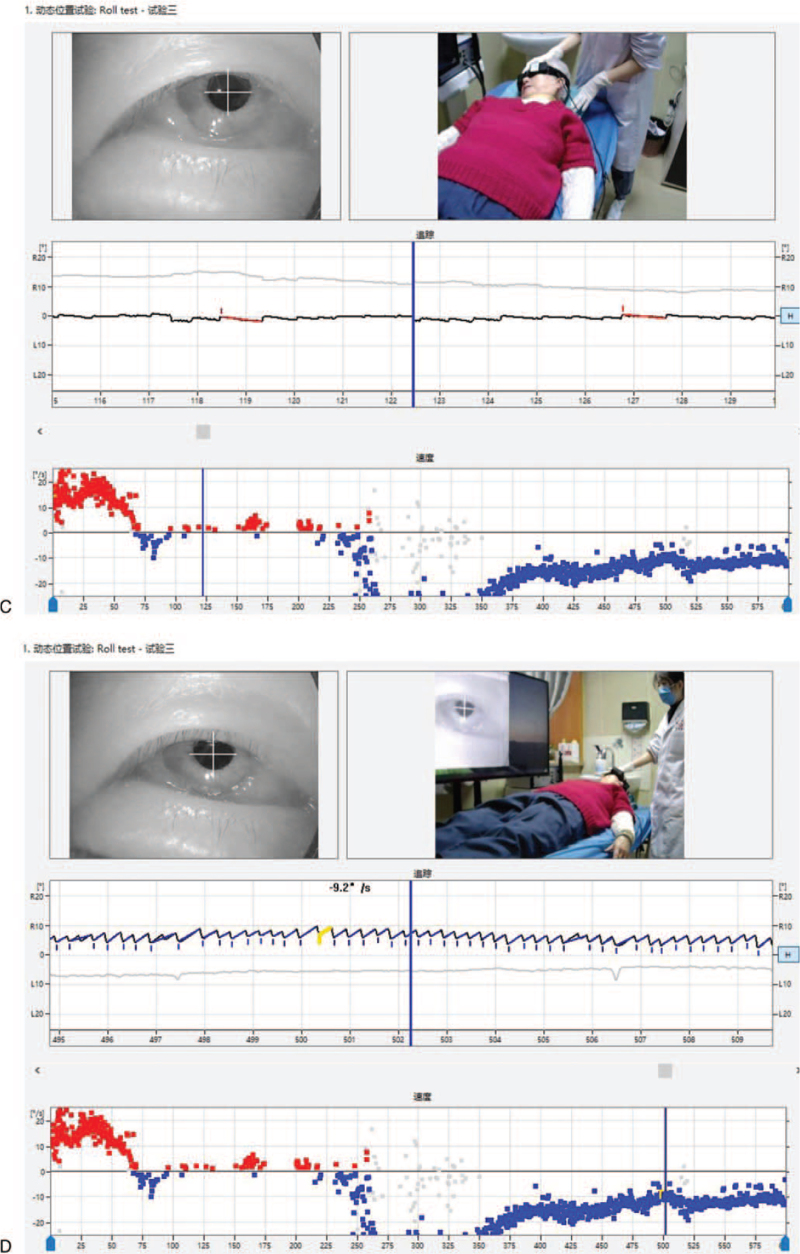
(A) A left horizontal nystagmus with an SPV of approximately 7.8°/s could be observed in the supine position. (B) In the Roll Test, a 90° rotation of the head to the right side induced sustained horizontal right nystagmus with an SPV of 24.2°/s. (C) With the head turned 15 to 20° to the right in the supine position, no nystagmus (second zero plane) was observed. (D) In the Roll Test, with the head turned 90° to the left, sustained horizontal left nystagmus was observed with an SPV of 9.2°/s. (E) A horizontal left nystagmus with an SPV of approximately 1.5°/s could be observed in the sitting position. (F) A horizontal right nystagmus with an SPV of 14.1°/s could be observed when the head was lowered 90° down in the sitting position. (G) The nystagmus disappeared (first zero plane) when the head was lowered 30° down in the sitting position. (H) A horizontal right nystagmus with an SPV of 22.6°/s could be observed in the prone position. (I) When the head was turned 15 to 30° to the right in the prone position, the direction of the nystagmus changed from right to left (reflecting the third zero plane). However, the actual plane of the nystagmus could not be found.

**Figure 1 (Continued):**
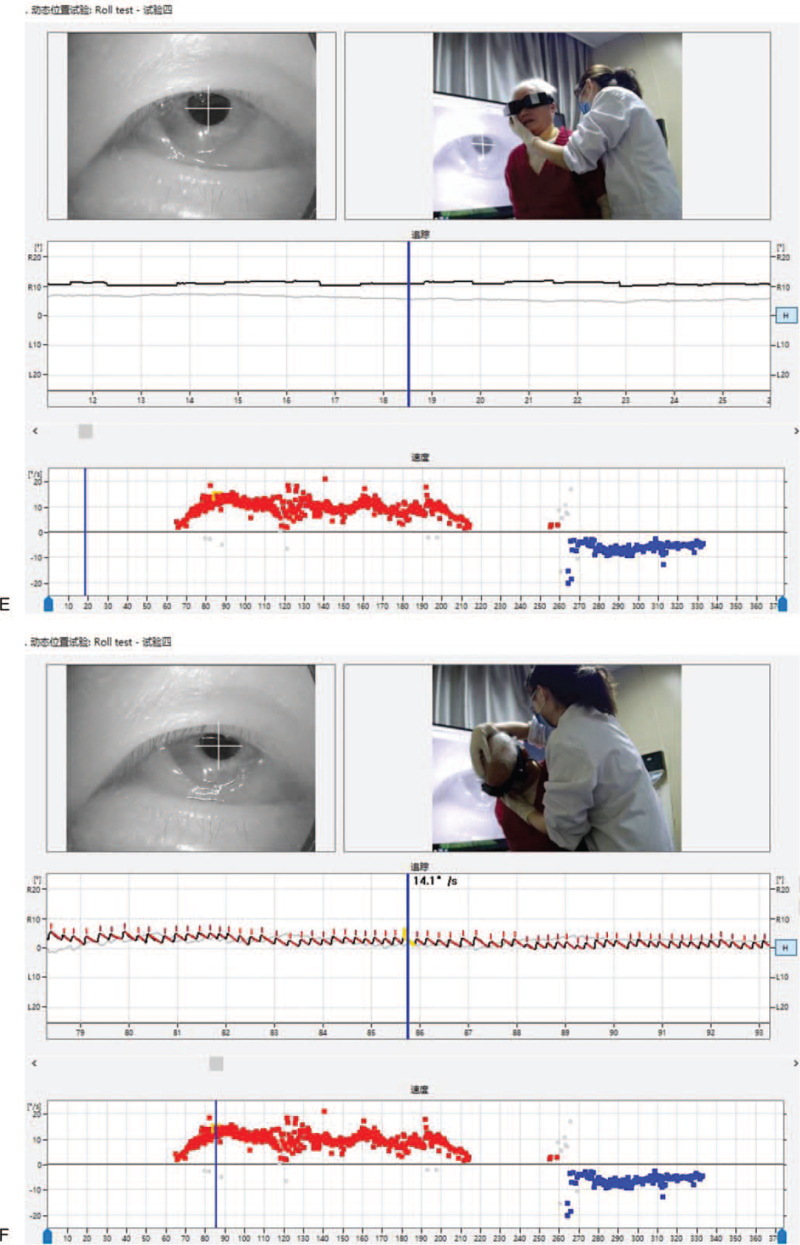
(A) A left horizontal nystagmus with an SPV of approximately 7.8°/s could be observed in the supine position. (B) In the Roll Test, a 90° rotation of the head to the right side induced sustained horizontal right nystagmus with an SPV of 24.2°/s. (C) With the head turned 15 to 20° to the right in the supine position, no nystagmus (second zero plane) was observed. (D) In the Roll Test, with the head turned 90° to the left, sustained horizontal left nystagmus was observed with an SPV of 9.2°/s. (E) A horizontal left nystagmus with an SPV of approximately 1.5°/s could be observed in the sitting position. (F) A horizontal right nystagmus with an SPV of 14.1°/s could be observed when the head was lowered 90° down in the sitting position. (G) The nystagmus disappeared (first zero plane) when the head was lowered 30° down in the sitting position. (H) A horizontal right nystagmus with an SPV of 22.6°/s could be observed in the prone position. (I) When the head was turned 15 to 30° to the right in the prone position, the direction of the nystagmus changed from right to left (reflecting the third zero plane). However, the actual plane of the nystagmus could not be found.

**Figure 1 (Continued):**
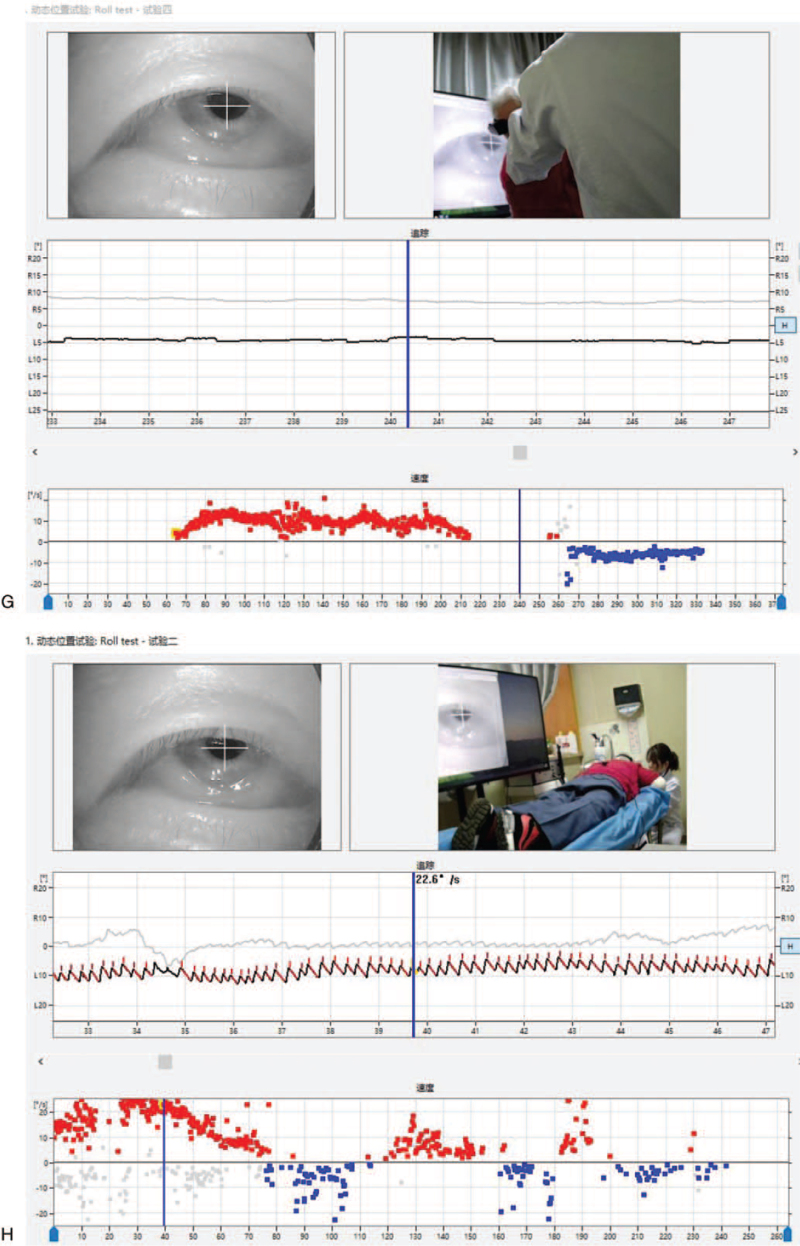
(A) A left horizontal nystagmus with an SPV of approximately 7.8°/s could be observed in the supine position. (B) In the Roll Test, a 90° rotation of the head to the right side induced sustained horizontal right nystagmus with an SPV of 24.2°/s. (C) With the head turned 15 to 20° to the right in the supine position, no nystagmus (second zero plane) was observed. (D) In the Roll Test, with the head turned 90° to the left, sustained horizontal left nystagmus was observed with an SPV of 9.2°/s. (E) A horizontal left nystagmus with an SPV of approximately 1.5°/s could be observed in the sitting position. (F) A horizontal right nystagmus with an SPV of 14.1°/s could be observed when the head was lowered 90° down in the sitting position. (G) The nystagmus disappeared (first zero plane) when the head was lowered 30° down in the sitting position. (H) A horizontal right nystagmus with an SPV of 22.6°/s could be observed in the prone position. (I) When the head was turned 15 to 30° to the right in the prone position, the direction of the nystagmus changed from right to left (reflecting the third zero plane). However, the actual plane of the nystagmus could not be found.

**Figure 1 (Continued):**
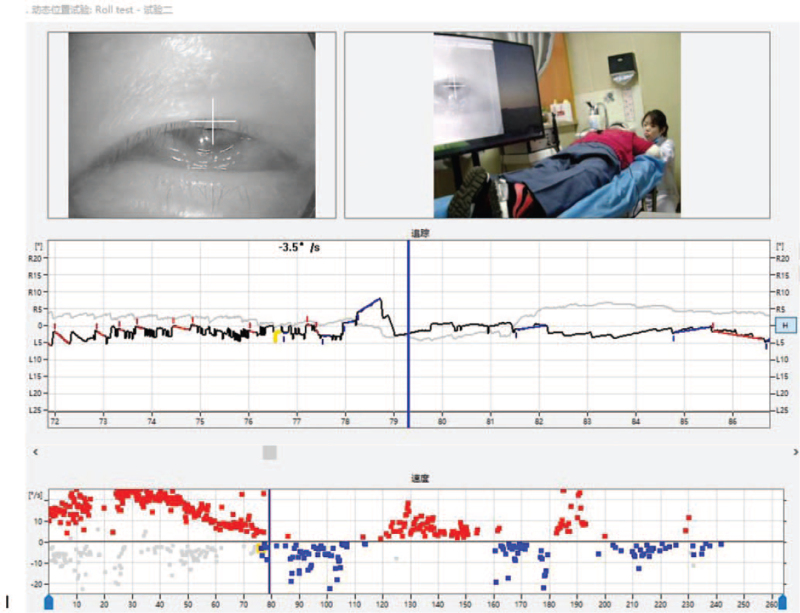
(A) A left horizontal nystagmus with an SPV of approximately 7.8°/s could be observed in the supine position. (B) In the Roll Test, a 90° rotation of the head to the right side induced sustained horizontal right nystagmus with an SPV of 24.2°/s. (C) With the head turned 15 to 20° to the right in the supine position, no nystagmus (second zero plane) was observed. (D) In the Roll Test, with the head turned 90° to the left, sustained horizontal left nystagmus was observed with an SPV of 9.2°/s. (E) A horizontal left nystagmus with an SPV of approximately 1.5°/s could be observed in the sitting position. (F) A horizontal right nystagmus with an SPV of 14.1°/s could be observed when the head was lowered 90° down in the sitting position. (G) The nystagmus disappeared (first zero plane) when the head was lowered 30° down in the sitting position. (H) A horizontal right nystagmus with an SPV of 22.6°/s could be observed in the prone position. (I) When the head was turned 15 to 30° to the right in the prone position, the direction of the nystagmus changed from right to left (reflecting the third zero plane). However, the actual plane of the nystagmus could not be found.

### 3.2 The bow test

1. A weak left horizontal nystagmus with an SPV of 1.5°/s could be observed when the patient was in the sitting position (Fig. 1    E).
2. The right horizontal nystagmus with an SPV of 14.1°/s could be observed when the patient was in the sitting position with her head down at 90°, although the intensity of the nystagmus gradually decreased (Fig. 1    F).
3. As the patient gradually raised her head to 30° down, the right nystagmus gradually weakened and disappeared (Fig. 1    G; first zero plane).

### 3.3 The lean test

1. The right horizontal nystagmus with an SPV of 22.6°/s could be observed when the patient was in the prone position (Fig. 1    H).
2. A turn of her head to the right could induce a gradual weakening of the horizontal right nystagmus. At 30°, a slight left nystagmus with a slow angular velocity SPV of 2°/s could be observed. The left nystagmus gradually increased as the patient continued to turn her head to the right (Fig. 1    I; third zero plane).

On the basis of the above examinations and displacement tests, all the diagnostic criteria of light cupula were met. In addition, all other vertigo-inducing diseases could be excluded. The patient was prescribed 25 mg of Difenidol hydrochloride tablets and 12 mg of Betahistine to be taken orally three times a day. At the telephone follow-up 1 month later, the patient reported that her vertigo symptoms had disappeared.

## 4 Discussion

Under normal conditions, the density of the endolymph is similar to that of the cupula, and the hair cells in the cupula are not activated or inhibited by a change in head orientation relative to gravity. However, when the density of the cupula is lower or higher than the density of the endolymph, the cupula may become sensitive to changes in head orientation. When the head is in upright position, the horizontal semicircular canal is high in the front and low in the back, and there is an angle of about 30° between the canal and the horizontal plane. The direction of growth of hair cell cilia in the horizontal semicircular canal is anteromedial oblique to the posterolateral, forming an angle of 30° with the anomalistic line. Ichijo reports that this anatomical angle is about 20° (with the posterior inner side as the ampulla side, and the anterior outer side as the tube side).

**Figure 2:**
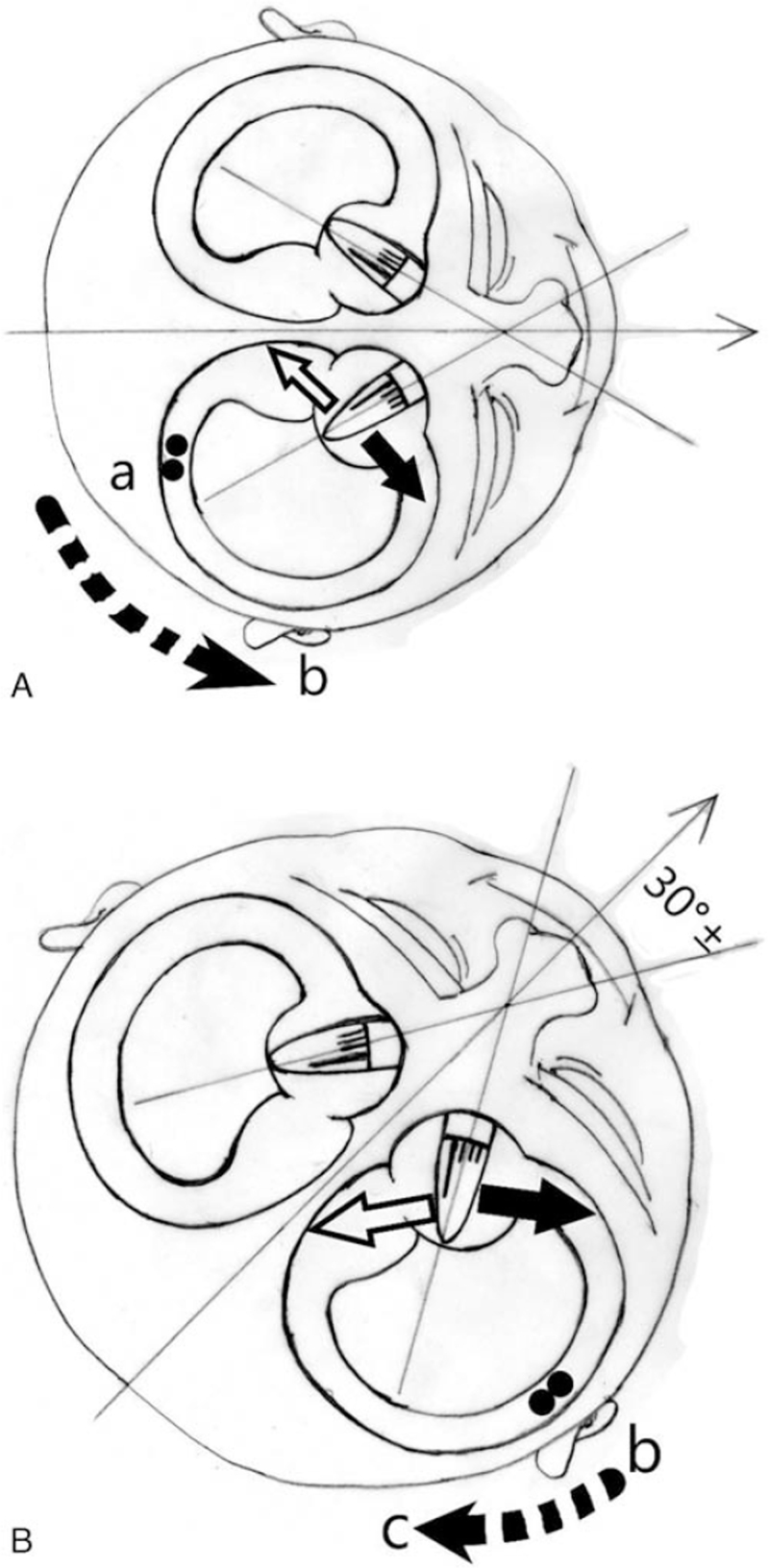
(A) Patients with cupula diseases. When a patient with cupula disease in the decubitus moves his head from right to left, the drift direction of the heavy cupula (black arrow) and light cupula (white arrow) does not change, and the direction of the nystagmus remains unchanged. Thus, the left nystagmus is observed with heavy cupula, and the right nystagmus is observed with light cupula. However, the angle made with the gravity line gradually decreases and the intensity of the nystagmus gradually changes. Patients with canalolithiasis. (B) Otoliths in a canalolithiasis patient move from point A to the ampulla to point B (dashed arrow), resulting in a right nystagmus. In the process of the patient turning his head from right to left, the otoliths move from point B to the ampulla to point C (dashed arrow), reversing the nystagmus (that is, producing the left nystagmus). As long as the flow speed remains unchanged, the intensity of the nystagmus remains unchanged.

Due to this anatomical feature, several effects are observed when the density of the cupula changes:

1. When the patient lowers their head by 30°, the first zero plane is formed. When the patient raises their head by 60° or tilts their head by 30° to the affected side in the supine position, the second zero plane is formed. When the patient lowers his head 90° or tilts his head 30° to the affected side in the prone position, the third zero plane is formed. The first zero plane is not a unique zero plane of cupula disease (this zero plane is also observed in patients with horizontal semicircular canalolithiasis). In contrast, the second and third zero planes are unique zero planes of cupula disease (these zero planes do not occur in canalolithiasis).
2. An opposite nystagmus is observed when the head is on either side of the zero plane. Due to anatomical variations in the human body, the angle of the zero plane may not be exactly equal to 30°. According to Ichijo, the anatomical angle is about 20° (the actual zero-plane angle is between 5° and 89°). It may be difficult to find the zero plane where the nystagmus completely disappears during clinical examination. Therefore, the existence of zero plane can be inferred from the observation of a reverse in the nystagmus near the zero plane.
3. If the angle of movement does not exceed the zero plane when the head is moved to 1 side (either the healthy side or the affected side of the zero plane), then the direction of the cupula pulling the hair cell cilia remains unchanged. Under these circumstances, the direction of the nystagmus also remains unchanged. However, if the angle of the long axis of the hair cell cilia relative to the deflection of the gravity vector plane changes, then the intensity of the nystagmus will change accordingly (although the direction of the nystagmus remains unchanged). In patients with otolithiasis, the opposite situation is observed. When the head is turned to one side, the nystagmus will change direction. Nevertheless, if we still keep a constant speed, the intensity of the nystagmus will remain the same even if the direction of movement is different, (Fig. 2A–B).
4. Excitatory nystagmus towards the affected side is caused by the real excitatory stimulus of the diseased semicircular canal, while pseudoexcitatory nystagmus towards the uninfected side is caused by inhibitory stimulation of the affected side. According to Ewald's law, the intensity of the excited nystagmus is greater than that of the inhibitory nystagmus. Therefore, theoretically speaking, the direction of stronger nystagmus in canalolithiasis and cupula disease should point to the affected side. In the 90° head-down or prone position, the hair cells of the light cupula drift to the ampulla side and produce excitatory stimulation. In contrast, when the head is in the 60° up or supine position, the hair cells of the light cupula drift to the side of the tube and produce inhibitory stimulation. The opposite is true for heavy crest caps for heavy cupula. Therefore, if there is a second or third zero plane, the nystagmus intensity observed during head down is greater than the nystagmus intensity observed during head up, and light cupula can be inferred (and vice versa).

Both the light cupula and the HC-BPPV present geotrophic DCPN, while both the heavy cupula and the horizontal semicircular canal forearm canalithasis present apogeotrophic DCPN. The expected duration of the nystagmus produced by HC-BPPV is less than 1 minutes, and the intensity of this nystagmus is expected to easily fatigue (to weaken or disappear). However, the duration of nystagmus of cupula disease is more than 1 minutes. We propose that the short duration of the nystagmus in HC-BPPV is not caused by the fatigue of the hair cells but by the time limit of otolith flow to the lowest point. If the number of otoliths in HC-BPPV is large (or if there is a stenosis in the semicircular canal), a ’hourglass effect’ may be created and the flow of the otoliths may be prolonged (to >1 minutes). If the otoliths are within the lumen of the horizontal semicircular canal, the otolith flow will continue as long as rotation of the head causes the otoliths to change their relative position. Under these circumstances, there may not be a definite incubation period.

In the present case, nystagmus was observed when the roll test, the bow test, and the prone position test were performed. While the duration of these nystagmus was relatively long, some attenuation was apparent. It can be concluded that nystagmus in cupula disease also shows fatigue. This fatigue in light cupula nystagmus has not been previously reported in the literature. However, if the duration and latency of nystagmus are not the most critical diagnostic criteria, then the presence of second and third zero planes and the occurrence of reversed nystagmus should be considered the most characteristic signs when the head is located on either side of the zero plane.

We can infer several facts from our observations of the patient in the present case study.

**Figure 3:**
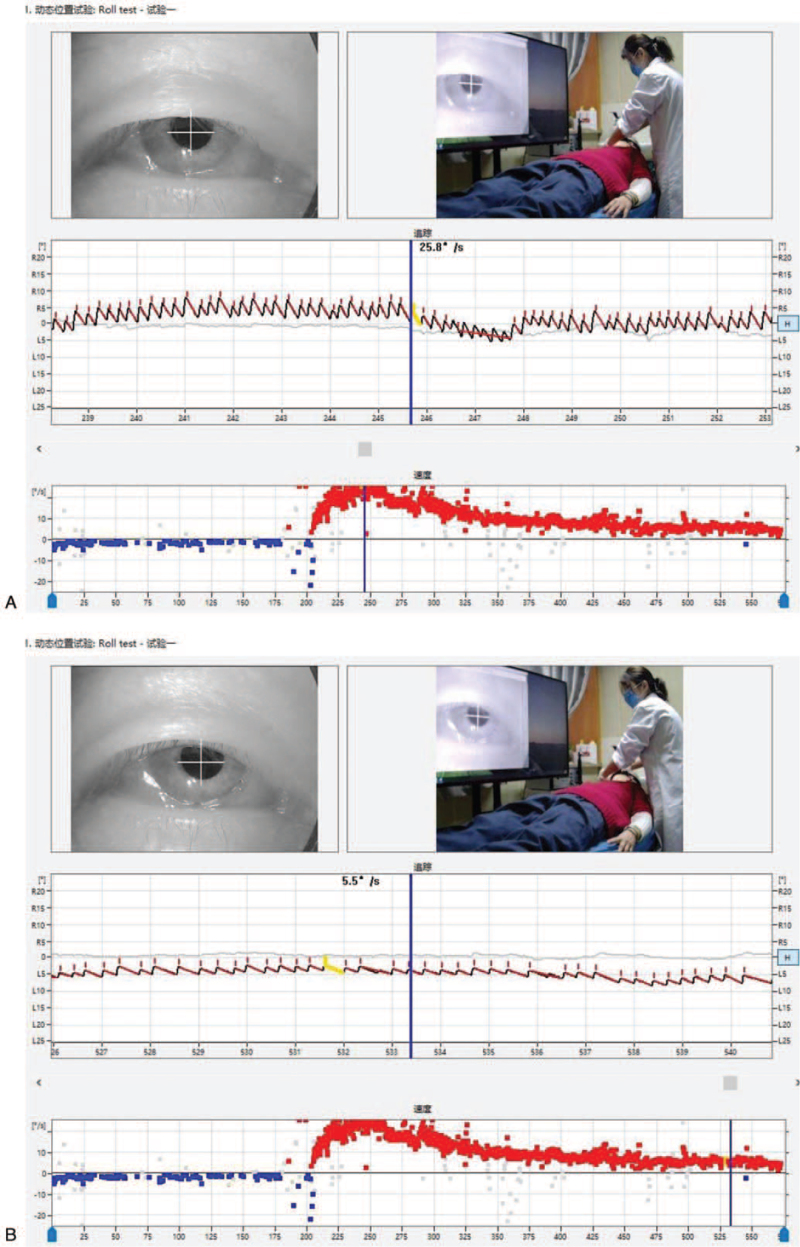
(A) The horizontal right nystagmus can be observed with the head turned 90° on the right side in the roll test. The right nystagmus (geotropic nystagmus) with no latency and long duration (>5 minutes) was recorded. (B) The SPV of the initial nystagmus was 5.5°/s and the maximum nystagmus (SPV of 25.8°/s) was reached after 40 second. The nystagmus then gradually decreased, reaching 7.0°/s after 5 minutes.

1. The first and second zero planes could be demonstrated in the patient. Although the position where the nystagmus completely disappeared was not found during head turning to the right in the prone position, the original right nystagmus changed to a slight left nystagmus when the head turned 30° to the right. Thus, a zero plane (the third zero plane) was present between these 2 head positions. From this observation, it can be inferred that the patient had cupula disease instead of canalolithiasis.
2. The patient presented left nystagmus in the sitting and supine positions, and right nystagmus in the head down and prone positions. Furthermore, the nystagmus in the prone position was greater than the nystagmus in the supine and sitting positions (that is, the intensity of the right nystagmus was greater than that of the left nystagmus). According to Ewald's law, the prone position is where the hair cell cilia deviate to the ventral side of the ampulla to produce greater nystagmus. Therefore, it can be inferred that the patient had a light cupula (not a heavy cupula) and the lesion was on the right side.
3. During the head down position, the prone position, and the roll test, when the patient moved his head on either side of the zero plane, it was found that while the direction of the nystagmus did not change (as long as the zero plane was not crossed), the intensity of the nystagmus did change. In the process of turning the head to the left in the right decubitus position (when the second zero plane was not crossed), the intensity of the right nystagmus gradually weakened. As the head crossed the zero plane, the nystagmus direction changed to the left. The left nystagmus then gradually weakened during the process of turning the head to the right from the left decubitus position to the midline.
4. Although the duration of the nystagmus (at several different positions) lasted for a comparatively long time (observation time > 5 minutes), some attenuation was apparent (Fig. 3A–B). Thus, it can be concluded that nystagmus in cupula disease also shows fatigue.

According to the “heavy endolymph” hypothesis, the heavier endolymph makes the cupula lighter. In a specific head position, the cupula pulls the hair cell cilia, causing them to drift to the ventral or tube side of the ampullae, resulting in PG-DCPN nystagmus. However, Kim proposed a new hypothesis in 2019. According to Kim, the light cupula may actually be caused by a higher specific gravity of exolymphatic fluid, rather than a higher specific gravity of endolymphatic fluid.^[17]^ In this “heavy exolymph” hypothesis, the increased density of the exolymphatic fluid causes the lighter endolymphatic fluid to float upward, which accounts for the PG-DCPN nystagmus. Kim and colleagues reported a case of light cupula that affected the three right semicircular canals. Kim suggested that changes in endolymphatic density could simultaneously affect the three semicircular canals on the same side. The resulting nystagmus demonstrated rotation and vertical components during the induction test. Several related studies were also reported.^[16]^

In the process of measuring the zero plane of patients with PG-DCPN, Ichijo noted that the actual zero plane varied greatly. Furthermore, he figured it was not exactly attributed to the anatomical variation but more likely, those differences in the location of particles adhering to the cupula.^[8]^ However, the heavy endolymph and heavy exolymph hypotheses cannot account for this variation, because they do not posit a role for diseased particles. Therefore, PG-DCPN may be caused by cupula disease, rather than changes in the specific gravity of the endolymph and exolymph.

In the present study, the zero plane angles of each body position during the induction test were roughly the same, which is consistent with the hypotheses of heavy endolymph and heavy exolymph. However, no significant rotation or vertical components of the nystagmus were observed, which is consistent with the hypothesis of granular disease. It remains possible that changes in the density of internal and external lymphatic fluid result in vertical and rotary nystagmus, and that these changes are masked by the strong horizontal nystagmus. It remains to be determined whether PG-DCPN is caused by changes in internal or external lymphatic density, granule disease, or a combination of both.

## Author contributions

**Conceptualization:** Juan-Juan Li, Ying Lin.

**Data analysis:** Yang Zhang, Juan-Juan Li.

**Data curation:** Juan-Juan Li.

**Data extraction:** Zhaoxia Wang, Qiang Guo.

**Final draft:** Yang Zhang, Juan-Juan Li.

**Initial draft:** Zhaoxia Wang, Yang Zhang.

**Literature search and review:** Zhaoxia Wang, Yang Zhang.

**Methodology:** Zhaoxia Wang, Qiang Guo.

**Writing – original draft:** Zhaoxia Wang.

**Writing – review & editing:** Yang Zhang, Juan-Juan Li.

## References

[1] Shigeno K Takahashi H. Static directionchanging horizontal positional nystagmus of peripheral origin. J Vest Res 2001;11:243–4.

[2] Seo T Shiraishi K Kobayashi T Mutsukazu K Doi K. Clinical course of persistent geotropic direction-changing positional nystagmus with neutral position-Light cupula. Acta Otolaryngol 2016;136:34–7. 26382554 10.3109/00016489.2015.1079926

[3] Kim YW Shin JE Lee YS Kim CH. Persistent positional vertigo in a patient with sudden sensorineural hearing loss: a case report. J Audiol Otol 2015;19:104–7. 26413578 10.7874/jao.2015.19.2.104 PMC4582458

[4] Ichijo H. Recurrence in patients with benign paroxysmal positional vertigo of the lateral semicircular canal. Auris Nasus Larynx 2020;47:353–8. 31753472 10.1016/j.anl.2019.10.008

[5] Hiruma K Numata T. Positional nystagmus showing neutral points. ORL J Otorhinolaryngol Relat Spec 2004;66:46–50. 15107588 10.1159/000077234

[6] Tomanovic T Bergenius J. Can the nystagmus pattern in patients with a ’light cupula’ be reproduced in hemi-labyrinthectomized subjects during positional alcohol nystagmus 1? Acta Otolaryngol 2011;131:929–36. 21563872 10.3109/00016489.2011.574645

[7] Kim CH Shin JE Kim YW. A new method for evaluating lateral semicircular canal cupulopathy. Laryngoscope 2015;125:1921–5. 25640211 10.1002/lary.25181

[8] Ichijo H. Neutral position of persistent direction-changing positional nystagmus. Eur Arch Otorhinolaryngol 2016;273:311–6. 25613295 10.1007/s00405-014-3487-3

[9] Schuknecht HF. Positional vertigo: clinical and experimental observations. Trans Am Acad Ophthalmol Otolaryngol 1962;66:319–32. 13909445

[10] Hiruma K Numata T Mitsuhashi T Tomemori T Watanabe R Okamoto Y. Two types of direction-changing positional nystagmus with neutral points. Auris Nasus Larynx 2011;38:46–51. 20724087 10.1016/j.anl.2010.07.004

[11] Ichijo H. Persistent direction-changing geotropic positional nystagmus. Eur Arch Otorhinolaryngol 2012;269:747–51. 21748654 10.1007/s00405-011-1700-1

[12] Naganawa S Ishihara S Iwano S Sone M Nakashima T. Detection of presumed hemorrhage in the ampullar endolymph of the semicircular canal: a case report. Magn Reson Med Sci 2009;8:187–91. 20035128 10.2463/mrms.8.187

[13] Kim CH Kim MB Ban JH. Persistent geotropic direction-changing positional nystagmus with a null plane: the light cupula. Laryngoscope 2014;124:E15–9. 24166487 10.1002/lary.24048

[14] Choi JY Lee ES Kim HJ Kim JS. Persistent geotropic positional nystagmus after meningitis: Evidence for light cupula. J Neurol Sci 2017;379:279–80. 28716260 10.1016/j.jns.2017.06.036

[15] Kim MB Hong SM Choi H. The light cupula: an emerging new concept for positional vertigo. J Audiol Otol 2018;22:01–5. 10.7874/jao.2017.00234 PMC5784369 29061034

[16] Kim CH Pham NC. Density difference between perilymph and endolymph: a new hypothesis for light cupula phenomenon. Med Hypotheses 2019;123:55–9. 30696592 10.1016/j.mehy.2018.12.017

[17] Kim CH Shin JE Shin DH Kim YW Ban JH. “Light cupula” involving all three semicircular canals: a frequently misdiagnosed disorder. Med Hypotheses 2014;83:541–4. 25249441 10.1016/j.mehy.2014.09.002

